# Development and Evaluation of the Content and Face Validity of an Mpox Questionnaire for Primary Healthcare Workers

**DOI:** 10.21315/mjms-01-2026-064

**Published:** 2026-04-30

**Authors:** Muhammad Saufi Abdullah, Suhaily Mohd Hairon, Siti Suhaila Mohd Yusoff, Mohd Azimullah Abdullah Zakaria, Mohd Shamril Mohd Nawi

**Affiliations:** 1 Department of Community Medicine, School of Medical Sciences, Universiti Sains Malaysia, Health Campus, Kubang Kerian, Kelantan, Malaysia; 2 Department of Family Medicine, School of Medical Sciences, Universiti Sains Malaysia, Health Campus, Kubang Kerian, Kelantan, Malaysia; 3 Communicable Disease Control Unit, Kelantan State Health Department, Kota Bharu, Kelantan, Malaysia; 4 Kota Kinabalu Area Health Office, Sabah State Health Department, Kota Kinabalu, Sabah, Malaysia

**Keywords:** mpox, questionnaire development, content validity, face validity, primary healthcare workers

## Abstract

**Background:**

Primary healthcare workers are essential in managing infectious diseases, including mpox. A validated instrument is required to assess their knowledge and readiness for mpox management. This study evaluated the content and face validity of a Malay-language mpox questionnaire for primary healthcare workers in Malaysia.

**Methods:**

Questionnaire development was conducted in two stages: i) item generation and ii) judgement and quantification. Ten experts from relevant disciplines assessed content validity, while 10 primary healthcare workers participated in face validation. The content validity index (CVI), face validity index (FVI), and modified Kappa were calculated to evaluate clarity, relevance, necessity, and representativeness.

**Results:**

The initial questionnaire comprised 62 items across two domains (knowledge and readiness). During content validation, one item assessing clarity yielded an item-level CVI (I-CVI) of 0.70 and a modified Kappa of 0.67; other items achieved excellent thresholds (I-CVI ≥ 0.78; modified Kappa ≥ 0.74). Scale-level CVI (S-CVI/Ave) across all criteria was >0.90, indicating excellent overall validity. The universal agreement S-CVI (S-CVI/UA) for relevance was 0.76, slightly below the recommended cut-off. Based on these findings and expert recommendations, one item was removed due to subthreshold performance and redundancy. Face validation demonstrated strong comprehensibility, with item-level FVI (I-FVI), scale-level FVI (S-FVI/Ave), and universal agreement S-FVI (S-FVI/UA) values exceeding recommended thresholds (0.78, 0.90, and 0.83, respectively). The final questionnaire contained 61 items.

**Conclusion:**

This study provides a validated Malay-language questionnaire for assessing knowledge and readiness for mpox management among primary healthcare workers. This tool provides a standardised approach for identifying knowledge gaps and guiding targeted training to strengthen outbreak preparedness.

## Introduction

Mpox is a zoonotic viral disease caused by the monkeypox virus, historically endemic to Central and West Africa (1). The first reported human case was documented in 1970 in the Democratic Republic of the Congo (2–4). Mpox garnered global public health attention following outbreaks in non-endemic regions beginning in May 2022 (5). By the end of 2024, more than 100,000 confirmed cases and over 220 deaths had been reported worldwide (6). The first confirmed mpox case in Malaysia was identified in July 2023 (7). By the end of 2025, 12 additional cases had been reported, bringing the cumulative total to 23 confirmed cases (8).

Primary healthcare workers constitute a critical frontline workforce during infectious disease outbreaks, as they are responsible for early detection, initial case management, and the implementation of infection prevention and control measures (9, 10). Knowledge encompasses awareness and understanding of mpox-related information, including transmission pathways, clinical manifestations, prevention strategies, and management protocols (11). Readiness reflects behavioural preparedness, integrating attitudes, social influences, perceived behavioural control, and intentions to undertake required preventive and control actions (12, 13). Gaps in knowledge or readiness may contribute to delayed diagnosis, suboptimal patient care, and heightened risk of transmission (14). Consequently, assessing these factors is essential for identifying specific gaps, strengthening the health system, and ensuring effective implementation of guidelines (15).

Developing a survey instrument requires rigorous validation to ensure that it accurately captures the intended constructs (16, 17). Questionnaires that are unvalidated or poorly designed may introduce measurement error, bias conclusions, and limit meaningful comparability across populations (18, 19). While numerous studies have examined mpox-related knowledge and attitudes among healthcare professionals globally, most have relied on instruments adapted from earlier studies without reporting comprehensive psychometric validation (20, 21). In Malaysia, surveys have been conducted among dental and medical students (10, 22); however, to our knowledge, no validated instrument exists to assess mpox-related knowledge and readiness among primary healthcare workers specifically. A validated Malay-language questionnaire is particularly important, as primary healthcare workers often serve as the first point of contact for suspected mpox cases in the community (5). Such a tool would facilitate systematic assessment of knowledge and readiness, identify training needs, guide targeted capacity-building, and provide a standardised approach for monitoring preparedness in Malaysian primary care settings (23). Moreover, the availability of a validated instrument could support future research and programme evaluation related to outbreak preparedness and response (18, 24, 25). This study addresses this gap by developing a Malay-language questionnaire and evaluating its content and face validity to ensure its suitability for primary healthcare workers in Malaysia.

## Methods

### Study Design

This cross-sectional study evaluated the content and face validity of a newly developed Malay-language mpox questionnaire for primary healthcare workers in Malaysia. Data collection was conducted from January to May 2025. Questionnaire development comprised two stages: item generation and validation. The first stage involved the systematic development of questionnaire items. This included establishing a clear purpose and rationale, identifying relevant constructs, and drafting items based on these constructs (26). The second stage involved expert and face validation to judge and quantify the relevance and clarity of the items (26).

### Clear Purpose and Rationale

A questionnaire should specify its intended outcome, target participants, justification for its development, and potential contribution to the field (26). This questionnaire was designed to assess the knowledge and readiness of primary healthcare workers in Malaysia regarding mpox management. The instrument provides a standardised measure to identify knowledge gaps, determine training needs, and inform targeted interventions to enhance outbreak response capacity.

### Identification of Constructs

A construct refers to the concept or domain that a questionnaire aims to measure (27). Key elements for inclusion were identified through a comprehensive literature review using relevant search keywords, including “monkeypox,” “knowledge,” “readiness,” “preparedness,” “healthcare workers,” and “questionnaire” (26). The questionnaire comprised two domains: knowledge and readiness. The knowledge domain was conceptually informed by the knowledge component of the knowledge-attitude-practice framework, in which knowledge serves as the foundation for subsequent attitudes and practices (18, 24, 28). The readiness domain was guided by the Theory of Planned Behaviour, particularly the constructs of attitude toward the behaviour, subjective norms, perceived behavioural control, and intention (12, 13). These theoretical frameworks, together with the literature review findings and relevant guidelines, informed construct identification and item development.

### Development of Items

An expert panel, comprising two public health medicine specialists and two family medicine specialists, developed the questionnaire items, ensuring clarity and comprehensibility for participants (16). Both local and international guidelines were consulted to enhance the accuracy and relevance of the items, minimise ambiguity, strengthen clarity, and optimise the quality of participants’ responses (29). The panel held a series of meetings to discuss, review, and refine items until consensus was reached on the draft questionnaire. The final instrument included 62 items across 2 domains. The knowledge domain comprised 36 items with response options of “true,” “false,” and “unsure,” scored as 1 for correct answers and 0 for incorrect and unsure responses. The readiness domain comprised 26 items, each rated on a 5-point Likert scale (“strongly disagree” = 1 to “strongly agree” = 5).

### Expert Validation

Ten experts from public health, family medicine, and infectious disease disciplines were appointed to evaluate the content validity of the questionnaire (Table 1). The recommended number of experts for such evaluations typically ranges from 6 to 10 (27). Experts assessed each item for clarity, relevance to the construct, necessity for inclusion, and representativeness in covering the construct (16). Each item was rated on a 4-point Likert scale. These ratings were used to calculate the content validity index (CVI) and the modified Kappa statistic. The item-level CVI (I-CVI) represents the proportion of experts assigning a score of 3 or 4 to an item. The scale-level CVI (S-CVI/Ave) is calculated as the average of the I-CVI scores across all items. The universal agreement S-CVI (S-CVI/UA) is calculated as the proportion of all items that were rated 3 or 4 by all experts (30). The modified Kappa (K) was calculated using the formula K = (I-CVI − Pc)/(1 − Pc), where Pc represents the probability of chance agreement among experts. Pc was computed as Pc = [N!/(A!(N-A)!)] × 0.5^N^, where N is the total number of experts, and A is the number of experts who rated the item as 3 or 4 (30). The recommended cut-offs for I-CVI, S-CVI/Ave, S-CVI/UA, and modified Kappa are ≥ 0.78, ≥ 0.90, ≥ 0.83, and ≥ 0.74, respectively (30).

### Face Validation

Three medical officers, two medical assistants, and five nurses from a government health clinic were recruited to participate in cognitive interviews (Table 2). Ten participants represent the minimum recommended number for such interviews (31). Participants were briefed on the procedure, and informed consent was obtained. Cognitive interviews were conducted using the think-aloud technique and verbal probing (16). Following the interviews, participants rated each item for clarity, relevance, necessity, and representativeness using a 4-point Likert scale. The face validity index (FVI) was then computed. Calculations and cut-off values for item-level FVI (I-FVI), scale-level FVI (S-FVI/Ave), and universal agreement S-FVI (S-FVI/UA) were analogous to those used for the CVI (27, 30).

## Results

### CVI and Modified Kappa

The content validity assessment demonstrated generally high agreement among experts across all four criteria (clarity, relevance, necessity, and representativeness) (Tables 3a and 3b). For clarity, the I-CVI ranged from 0.70 to 1.00, with one item, K08, falling below the recommended cut-off (I-CVI = 0.70). The scale-level index was high (S-CVI/Ave = 0.98), while the universal agreement was slightly below the recommended threshold (S-CVI/UA = 0.82). Modified Kappa for clarity ranged from 0.66 to 1.00, with K08 again below the recommended cut-off (K = 0.66).

For relevance, the I-CVI values ranged from 0.80 to 1.00, and the scale-level index remained high (S-CVI/Ave = 0.97). However, universal agreement was below the recommended threshold (S-CVI/UA = 0.76). Modified Kappa values ranged from 0.79 to 1.00, indicating excellent expert agreement. For both necessity and representativeness, I-CVI values also ranged from 0.80 to 1.00, with high scale-level indices (S-CVI/Ave = 0.98) and satisfactory universal agreement (S-CVI/UA = 0.85). Corresponding modified Kappa values ranged from 0.79 to 1.00, suggesting excellent agreement among experts.

Item K08 did not meet the recommended thresholds for I-CVI or modified Kappa for clarity. Expert feedback indicated that it was redundant with item K07d. Consequently, based on statistical evidence and expert recommendations, item K08 was removed due to below-threshold values and redundancy.

### FVI

Face validity assessment revealed excellent agreement across clarity, relevance, necessity, and representativeness (Table 4). The I-FVI was 1.00 for all items, and the scale-level index (S-FVI/Ave) was also 1.00. Universal agreement was consistently high across all criteria (S-FVI/UA = 0.98). These indices met or exceeded recommended thresholds, supporting an acceptable level of response process validity. As all items exceeded the minimum recommended thresholds, no further modifications were required. The final questionnaire retained 61 items.

## Discussion

The findings of this study indicate that the questionnaire demonstrates excellent content and face validity, supporting its suitability for the target population. Regarding content validity, the expert panel demonstrated high agreement across the clarity, relevance, necessity, and representativeness criteria. The S-CVI/Ave for all criteria exceeded the recommended threshold of 0.90, indicating excellent overall content validity (27, 30). Item K08 was identified as an outlier for clarity, with an I-CVI of 0.70 and a modified Kappa of 0.67, both below the recommended cut-offs of 0.78 and 0.74, respectively (27, 30). However, relying on validity indices alone is often insufficient for comprehensive instrument refinement (32). The decision to eliminate item K08 was further supported by expert feedback, which indicated redundancy with item K07d. Content validity involves not only the inclusion of essential items but also the removal of undesirable or repetitive items to ensure instrument parsimony (33). Consequently, the low validity indices, combined with expert consensus on redundancy, provided strong justification for deleting item K08. This approach aligns with best practices in instrument validation, which recommend integrating expert comments with quantitative scoring to refine the domain and ensure that the final instrument is both statistically robust and conceptually clear (27, 30).

The S-CVI/UA for relevance was 0.76, slightly below the recommended threshold of 0.83 (30). This lower value indicates that, while the average consensus was high, complete unanimity among the 10 experts was not achieved for all items. This result highlighted the need for necessary refinement, prompting the revision of item K08 to better align with the construct before face validation. The use of modified Kappa in this study was particularly important, as it adjusts the CVI for chance agreement, providing a more robust measure of expert consensus than proportion agreement alone (27, 30).

The face validity assessment yielded strong evidence of the instrument’s comprehensibility among the target population. The I-FVI results showed perfect agreement (I-FVI = 1.00) across all items for clarity, relevance, necessity, and representativeness, with S-FVI/Ave of 1.00 and S-FVI/UA of 0.98, exceeding the acceptable cut-offs for a panel of 10 raters (34). These findings suggest that the rigorous content validation and subsequent revisions were effective. The inclusion of cognitive interviews utilising think-aloud techniques and verbal probing provided substantial evidence of response process validity (16, 31), ensuring that the items were interpreted by medical officers, medical assistants, and nurses as intended by the developers, thereby minimising the risk of misinterpretation in field settings.

This study incorporated a rigorous validation process, involving a diverse panel of experts from relevant disciplines and the use of modified Kappa to ensure reliable expert consensus (30). Conducting cognitive interviews with a representative sample of the target population further strengthened evidence of response process validity (16). However, the validation was limited to content and face validity, which do not assess the instrument’s internal consistency. Therefore, future research should focus on additional validation steps to fully establish the psychometric properties of this questionnaire.

## Conclusion

This study successfully developed a Malay-language mpox questionnaire for primary healthcare workers and established its content and face validity. This validated questionnaire addresses a critical gap by providing a culturally and linguistically appropriate tool tailored to the Malaysian healthcare context. The instrument offers a foundational means for assessing the knowledge and readiness of primary healthcare workers, thereby facilitating the identification of training needs and the development of effective public health strategies for mpox management. Further psychometric evaluation involving large-scale field testing is recommended to confirm the instrument’s construct validity and reliability.

## Figures and Tables

**Table 1:** Sociodemographic characteristics of experts on the content validity of the questionnaire

Expert	Designation	Expertise	Experience (years)
1	Public health medicine specialist	Public health	> 10
2	Public health medicine specialist	Public health	> 10
3	Public health medicine specialist	Public health	> 10
4	Family medicine specialist	Family medicine	> 10
5	Family medicine specialist	Family medicine	> 10
6	Family medicine specialist	Family medicine	> 10
7	Family medicine specialist	Family medicine	> 10
8	Internal medicine specialist	Infectious disease	> 10
9	Internal medicine specialist	Infectious disease	> 10
10	Internal medicine specialist	Infectious disease	> 10

**Table 2:** Sociodemographic characteristics of participants on the face validity of the questionnaire

Participant	Occupation	Age (years)	Experience (years)
1	Medical officer	46	20
2	Medical officer	38	10
3	Medical officer	37	11
4	Assistant medical officer	54	28
5	Assistant medical officer	50	28
6	Nurse	39	17
7	Nurse	44	22
8	Nurse	40	15
9	Nurse	50	25
10	Nurse	54	28

**Table 3a:** CVI and modified Kappa of the mpox questionnaire for primary healthcare workers: clarity and relevance

Item	Clarity				Relevance			
	
	Agreement	I-CVI	Pc	K	Agreement	I-CVI	Pc	K
K01	10/10	1.00	9.77 × 10^−4^	1.00	10/10	1.00	9.77 × 10^−4^	1.00
K02	10/10	1.00	9.77 × 10^−4^	1.00	10/10	1.00	9.77 × 10^−4^	1.00
K03	9/10	0.90	9.77 × 10^−3^	0.90	9/10	0.90	9.77 × 10^−3^	0.90
K04	8/10	0.80	4.39 × 10^−2^	0.79	10/10	1.00	9.77 × 10^−4^	1.00
K05	10/10	1.00	9.77 × 10^−4^	1.00	10/10	1.00	9.77 × 10^−4^	1.00
K06	10/10	1.00	9.77 × 10^−4^	1.00	10/10	1.00	9.77 × 10^−4^	1.00
K07a	10/10	1.00	9.77 × 10^−4^	1.00	10/10	1.00	9.77 × 10^−4^	1.00
K07b	10/10	1.00	9.77 × 10^−4^	1.00	10/10	1.00	9.77 × 10^−4^	1.00
K07c	10/10	1.00	9.77 × 10^−4^	1.00	10/10	1.00	9.77 × 10^−4^	1.00
K07d	10/10	1.00	9.77 × 10^−4^	1.00	10/10	1.00	9.77 × 10^−4^	1.00
K07e	10/10	1.00	9.77 × 10^−4^	1.00	10/10	1.00	9.77 × 10^−4^	1.00
K08	7/10	0.70	1.17 × 10^−1^	0.66	8/10	0.80	4.39 × 10^−2^	0.79
K09	10/10	1.00	9.77 × 10^−4^	1.00	10/10	1.00	9.77 × 10^−4^	1.00
K10	9/10	0.90	9.77 × 10^−3^	0.90	9/10	0.90	9.77 × 10^−3^	0.90
K11	9/10	0.90	9.77 × 10^−3^	0.90	9/10	0.90	9.77 × 10^−3^	0.90
K12a	10/10	1.00	9.77 × 10^−4^	1.00	10/10	1.00	9.77 × 10^−4^	1.00
K12b	10/10	1.00	9.77 × 10^−4^	1.00	10/10	1.00	9.77 × 10^−4^	1.00
K12c	10/10	1.00	9.77 × 10^−4^	1.00	10/10	1.00	9.77 × 10^−4^	1.00
K13a	10/10	1.00	9.77 × 10^−4^	1.00	10/10	1.00	9.77 × 10^−4^	1.00
K13b	10/10	1.00	9.77 × 10^−4^	1.00	10/10	1.00	9.77 × 10^−4^	1.00
K13c	10/10	1.00	9.77 × 10^−4^	1.00	10/10	1.00	9.77 × 10^−4^	1.00
K13d	10/10	1.00	9.77 × 10^−4^	1.00	10/10	1.00	9.77 × 10^−4^	1.00
K14	10/10	1.00	9.77 × 10^−4^	1.00	9/10	0.90	9.77 × 10^−3^	0.90
K15	10/10	1.00	9.77 × 10^−4^	1.00	10/10	1.00	9.77 × 10^−4^	1.00
K16	10/10	1.00	9.77 × 10^−4^	1.00	10/10	1.00	9.77 × 10^−4^	1.00
K17	10/10	1.00	9.77 × 10^−4^	1.00	10/10	1.00	9.77 × 10^−4^	1.00
K18	10/10	1.00	9.77 × 10^−4^	1.00	10/10	1.00	9.77 × 10^−4^	1.00
K19	10/10	1.00	9.77 × 10^−4^	1.00	10/10	1.00	9.77 × 10^−4^	1.00
K20	10/10	1.00	9.77 × 10^−4^	1.00	9/10	0.90	9.77 × 10^−3^	0.90
K21	10/10	1.00	9.77 × 10^−4^	1.00	9/10	0.90	9.77 × 10^−3^	0.90
K22	10/10	1.00	9.77 × 10^−4^	1.00	10/10	1.00	9.77 × 10^−4^	1.00
K23	9/10	0.90	9.77 × 10^−3^	0.90	9/10	0.90	9.77 × 10^−3^	0.90
K24	10/10	1.00	9.77 × 10^−4^	1.00	9/10	0.90	9.77 × 10^−3^	0.90
K25	10/10	1.00	9.77 × 10^−4^	1.00	10/10	1.00	9.77 × 10^−4^	1.00
K26	9/10	0.90	9.77 × 10^−3^	0.90	9/10	0.90	9.77 × 10^−3^	0.90
K27	10/10	1.00	9.77 × 10^−4^	1.00	10/10	1.00	9.77 × 10^−4^	1.00
R01	10/10	1.00	9.77 × 10^−4^	1.00	10/10	1.00	9.77 × 10^−4^	1.00
R02	9/10	0.90	9.77 × 10^−3^	0.90	9/10	0.90	9.77 × 10^−3^	0.90
R03	9/10	0.90	9.77 × 10^−3^	0.90	10/10	1.00	9.77 × 10^−4^	1.00
R04	10/10	1.00	9.77 × 10^−4^	1.00	10/10	1.00	9.77 × 10^−4^	1.00
R05	10/10	1.00	9.77 × 10^−4^	1.00	10/10	1.00	9.77 × 10^−4^	1.00
R06	10/10	1.00	9.77 × 10^−4^	1.00	9/10	0.90	9.77 × 10^−3^	0.90
R07	10/10	1.00	9.77 × 10^−4^	1.00	10/10	1.00	9.77 × 10^−4^	1.00
R08	10/10	1.00	9.77 × 10^−4^	1.00	10/10	1.00	9.77 × 10^−4^	1.00
R09	10/10	1.00	9.77 × 10^−4^	1.00	10/10	1.00	9.77 × 10^−4^	1.00
R10	10/10	1.00	9.77 × 10^−4^	1.00	10/10	1.00	9.77 × 10^−4^	1.00
R11	10/10	1.00	9.77 × 10^−4^	1.00	10/10	1.00	9.77 × 10^−4^	1.00
R12	10/10	1.00	9.77 × 10^−4^	1.00	10/10	1.00	9.77 × 10^−4^	1.00
R13	10/10	1.00	9.77 × 10^−4^	1.00	10/10	1.00	9.77 × 10^−4^	1.00
R14	10/10	1.00	9.77 × 10^−4^	1.00	10/10	1.00	9.77 × 10^−4^	1.00
R15	10/10	1.00	9.77 × 10^−4^	1.00	10/10	1.00	9.77 × 10^−4^	1.00
R16	10/10	1.00	9.77 × 10^−4^	1.00	10/10	1.00	9.77 × 10^−4^	1.00
R17	10/10	1.00	9.77 × 10^−4^	1.00	10/10	1.00	9.77 × 10^−4^	1.00
R18	9/10	0.90	9.77 × 10^−3^	0.90	9/10	0.90	9.77 × 10^−3^	0.90
R19	10/10	1.00	9.77 × 10^−4^	1.00	10/10	1.00	9.77 × 10^−4^	1.00
R20	10/10	1.00	9.77 × 10^−4^	1.00	10/10	1.00	9.77 × 10^−4^	1.00
R21	10/10	1.00	9.77 × 10^−4^	1.00	10/10	1.00	9.77 × 10^−4^	1.00
R22	9/10	0.90	9.77 × 10^−3^	0.90	8/10	0.80	4.39 × 10^−2^	0.79
R23	10/10	1.00	9.77 × 10^−4^	1.00	10/10	1.00	9.77 × 10^−4^	1.00
R24	10/10	1.00	9.77 × 10^−4^	1.00	10/10	1.00	9.77 × 10^−4^	1.00
R25	10/10	1.00	9.77 × 10^−4^	1.00	10/10	1.00	9.77 × 10^−4^	1.00
R26	10/10	1.00	9.77 × 10^−4^	1.00	9/10	0.90	9.77 × 10^−3^	0.90
S-CVI/Ave		0.98				0.97		
S-CVI/UA		0.82				0.76		

Pc = probability of chance agreement; K = modified Kappa

**Table 3b:** CVI and modified Kappa of the mpox questionnaire for primary healthcare workers: necessity and representativeness

Item	Necessity				Representativeness			
	
	Agreement	I-CVI	Pc	K	Agreement	I-CVI	Pc	K
K01	10/10	1.00	9.77 × 10^−4^	1.00	10/10	1.00	9.77 × 10^−4^	1.00
K02	10/10	1.00	9.77 × 10^−4^	1.00	10/10	1.00	9.77 × 10^−4^	1.00
K03	9/10	0.90	9.77 × 10^−3^	0.90	9/10	0.90	9.77 × 10^−3^	0.90
K04	10/10	1.00	9.77 × 10^−4^	1.00	10/10	1.00	9.77 × 10^−4^	1.00
K05	10/10	1.00	9.77 × 10^−4^	1.00	10/10	1.00	9.77 × 10^−4^	1.00
K06	10/10	1.00	9.77 × 10^−4^	1.00	10/10	1.00	9.77 × 10^−4^	1.00
K07a	10/10	1.00	9.77 × 10^−4^	1.00	10/10	1.00	9.77 × 10^−4^	1.00
K07b	10/10	1.00	9.77 × 10^−4^	1.00	10/10	1.00	9.77 × 10^−4^	1.00
K07c	10/10	1.00	9.77 × 10^−4^	1.00	10/10	1.00	9.77 × 10^−4^	1.00
K07d	10/10	1.00	9.77 × 10^−4^	1.00	10/10	1.00	9.77 × 10^−4^	1.00
K07e	10/10	1.00	9.77 × 10^−4^	1.00	10/10	1.00	9.77 × 10^−4^	1.00
K08	8/10	0.80	4.39 × 10^−2^	0.79	8/10	0.80	4.39 × 10^−2^	0.79
K09	10/10	1.00	9.77 × 10^−4^	1.00	10/10	1.00	9.77 × 10^−4^	1.00
K10	9/10	0.90	9.77 × 10^−3^	0.90	9/10	0.90	9.77 × 10^−3^	0.90
K11	9/10	0.90	9.77 × 10^−3^	0.90	9/10	0.90	9.77 × 10^−3^	0.90
K12a	10/10	1.00	9.77 × 10^−4^	1.00	10/10	1.00	9.77 × 10^−4^	1.00
K12b	10/10	1.00	9.77 × 10^−4^	1.00	10/10	1.00	9.77 × 10^−4^	1.00
K12c	10/10	1.00	9.77 × 10^−4^	1.00	10/10	1.00	9.77 × 10^−4^	1.00
K13a	10/10	1.00	9.77 × 10^−4^	1.00	10/10	1.00	9.77 × 10^−4^	1.00
K13b	10/10	1.00	9.77 × 10^−4^	1.00	10/10	1.00	9.77 × 10^−4^	1.00
K13c	10/10	1.00	9.77 × 10^−4^	1.00	10/10	1.00	9.77 × 10^−4^	1.00
K13d	10/10	1.00	9.77 × 10^−4^	1.00	10/10	1.00	9.77 × 10^−4^	1.00
K14	10/10	1.00	9.77 × 10^−4^	1.00	10/10	1.00	9.77 × 10^−4^	1.00
K15	10/10	1.00	9.77 × 10^−4^	1.00	10/10	1.00	9.77 × 10^−4^	1.00
K16	10/10	1.00	9.77 × 10^−4^	1.00	10/10	1.00	9.77 × 10^−4^	1.00
K17	10/10	1.00	9.77 × 10^−4^	1.00	10/10	1.00	9.77 × 10^−4^	1.00
K18	10/10	1.00	9.77 × 10^−4^	1.00	10/10	1.00	9.77 × 10^−4^	1.00
K19	10/10	1.00	9.77 × 10^−4^	1.00	10/10	1.00	9.77 × 10^−4^	1.00
K20	10/10	1.00	9.77 × 10^−4^	1.00	10/10	1.00	9.77 × 10^−4^	1.00
K21	10/10	1.00	9.77 × 10^−4^	1.00	10/10	1.00	9.77 × 10^−4^	1.00
K22	10/10	1.00	9.77 × 10^−4^	1.00	10/10	1.00	9.77 × 10^−4^	1.00
K23	10/10	1.00	9.77 × 10^−4^	1.00	10/10	1.00	9.77 × 10^−4^	1.00
K24	10/10	1.00	9.77 × 10^−4^	1.00	10/10	1.00	9.77 × 10^−4^	1.00
K25	10/10	1.00	9.77 × 10^−4^	1.00	10/10	1.00	9.77 × 10^−4^	1.00
K26	9/10	0.90	9.77 × 10^−3^	0.90	9/10	0.90	9.77 × 10^−3^	0.90
K27	10/10	1.00	9.77 × 10^−4^	1.00	10/10	1.00	9.77 × 10^−4^	1.00
R01	10/10	1.00	9.77 × 10^−4^	1.00	10/10	1.00	9.77 × 10^−4^	1.00
R02	9/10	0.90	9.77 × 10^−3^	0.90	9/10	0.90	9.77 × 10^−3^	0.90
R03	10/10	1.00	9.77 × 10^−4^	1.00	10/10	1.00	9.77 × 10^−4^	1.00
R04	10/10	1.00	9.77 × 10^−4^	1.00	10/10	1.00	9.77 × 10^−4^	1.00
R05	10/10	1.00	9.77 × 10^−4^	1.00	10/10	1.00	9.77 × 10^−4^	1.00
R06	10/10	1.00	9.77 × 10^−4^	1.00	10/10	1.00	9.77 × 10^−4^	1.00
R07	10/10	1.00	9.77 × 10^−4^	1.00	10/10	1.00	9.77 × 10^−4^	1.00
R08	10/10	1.00	9.77 × 10^−4^	1.00	10/10	1.00	9.77 × 10^−4^	1.00
R09	10/10	1.00	9.77 × 10^−4^	1.00	10/10	1.00	9.77 × 10^−4^	1.00
R10	10/10	1.00	9.77 × 10^−4^	1.00	10/10	1.00	9.77 × 10^−4^	1.00
R11	10/10	1.00	9.77 × 10^−4^	1.00	10/10	1.00	9.77 × 10^−4^	1.00
R12	10/10	1.00	9.77 × 10^−4^	1.00	10/10	1.00	9.77 × 10^−4^	1.00
R13	10/10	1.00	9.77 × 10^−4^	1.00	10/10	1.00	9.77 × 10^−4^	1.00
R14	10/10	1.00	9.77 × 10^−4^	1.00	10/10	1.00	9.77 × 10^−4^	1.00
R15	10/10	1.00	9.77 × 10^−4^	1.00	10/10	1.00	9.77 × 10^−4^	1.00
R16	10/10	1.00	9.77 × 10^−4^	1.00	10/10	1.00	9.77 × 10^−4^	1.00
R17	10/10	1.00	9.77 × 10^−4^	1.00	10/10	1.00	9.77 × 10^−4^	1.00
R18	9/10	0.90	9.77 × 10^−3^	0.90	9/10	0.90	9.77 × 10^−3^	0.90
R19	10/10	1.00	9.77 × 10^−4^	1.00	10/10	1.00	9.77 × 10^−4^	1.00
R20	10/10	1.00	9.77 × 10^−4^	1.00	10/10	1.00	9.77 × 10^−4^	1.00
R21	10/10	1.00	9.77 × 10^−4^	1.00	10/10	1.00	9.77 × 10^−4^	1.00
R22	8/10	0.80	4.39 × 10^−2^	0.79	8/10	0.80	4.39 × 10^−2^	0.79
R23	10/10	1.00	9.77 × 10^−4^	1.00	10/10	1.00	9.77 × 10^−4^	1.00
R24	10/10	1.00	9.77 × 10^−4^	1.00	10/10	1.00	9.77 × 10^−4^	1.00
R25	10/10	1.00	9.77 × 10^−4^	1.00	10/10	1.00	9.77 × 10^−4^	1.00
R26	9/10	0.90	9.77 × 10^−3^	0.90	9/10	0.90	9.77 × 10^−3^	0.90
S-CVI/Ave		0.98				0.98		
S-CVI/UA		0.85				0.85		

Pc = probability of chance agreement; K = modified Kappa

**Table 4:** FVI of the mpox questionnaire for primary healthcare workers

Item	Clarity		Relevance		Necessity		Representativeness	
			
	Agreement	I-FVI	Agreement	I-FVI	Agreement	I-FVI	Agreement	I-FVI
K01	10/10	1.00	10/10	1.00	10/10	1.00	10/10	1.00
K02	10/10	1.00	10/10	1.00	10/10	1.00	10/10	1.00
K03	10/10	1.00	10/10	1.00	10/10	1.00	10/10	1.00
K04	10/10	1.00	10/10	1.00	10/10	1.00	10/10	1.00
K05	10/10	1.00	10/10	1.00	10/10	1.00	10/10	1.00
K06	10/10	1.00	10/10	1.00	10/10	1.00	10/10	1.00
K07a	10/10	1.00	10/10	1.00	10/10	1.00	10/10	1.00
K07b	10/10	1.00	10/10	1.00	10/10	1.00	10/10	1.00
K07c	10/10	1.00	10/10	1.00	10/10	1.00	10/10	1.00
K07d	10/10	1.00	10/10	1.00	10/10	1.00	10/10	1.00
K07e	10/10	1.00	10/10	1.00	10/10	1.00	10/10	1.00
K08	10/10	1.00	10/10	1.00	10/10	1.00	10/10	1.00
K09	10/10	1.00	10/10	1.00	10/10	1.00	10/10	1.00
K10	10/10	1.00	10/10	1.00	10/10	1.00	10/10	1.00
K11a	10/10	1.00	10/10	1.00	10/10	1.00	10/10	1.00
K11b	10/10	1.00	10/10	1.00	10/10	1.00	10/10	1.00
K11c	10/10	1.00	10/10	1.00	10/10	1.00	10/10	1.00
K12a	10/10	1.00	10/10	1.00	10/10	1.00	10/10	1.00
K12b	10/10	1.00	10/10	1.00	10/10	1.00	10/10	1.00
K12c	10/10	1.00	10/10	1.00	10/10	1.00	10/10	1.00
K12d	10/10	1.00	10/10	1.00	10/10	1.00	10/10	1.00
K13	10/10	1.00	10/10	1.00	10/10	1.00	10/10	1.00
K14	10/10	1.00	10/10	1.00	10/10	1.00	10/10	1.00
K15	10/10	1.00	10/10	1.00	10/10	1.00	10/10	1.00
K16	10/10	1.00	10/10	1.00	10/10	1.00	10/10	1.00
K17	10/10	1.00	10/10	1.00	10/10	1.00	10/10	1.00
K18	10/10	1.00	10/10	1.00	10/10	1.00	10/10	1.00
K19	10/10	1.00	10/10	1.00	10/10	1.00	10/10	1.00
K20	10/10	1.00	10/10	1.00	10/10	1.00	10/10	1.00
K21	10/10	1.00	10/10	1.00	10/10	1.00	10/10	1.00
K22	10/10	1.00	10/10	1.00	10/10	1.00	10/10	1.00
K23	10/10	1.00	10/10	1.00	10/10	1.00	10/10	1.00
K24	10/10	1.00	10/10	1.00	10/10	1.00	10/10	1.00
K25	10/10	1.00	10/10	1.00	10/10	1.00	10/10	1.00
K26	10/10	1.00	10/10	1.00	10/10	1.00	10/10	1.00
R01	10/10	1.00	10/10	1.00	10/10	1.00	10/10	1.00
R02	10/10	1.00	10/10	1.00	10/10	1.00	10/10	1.00
R03	10/10	1.00	10/10	1.00	10/10	1.00	10/10	1.00
R04	10/10	1.00	10/10	1.00	10/10	1.00	10/10	1.00
R05	10/10	1.00	10/10	1.00	10/10	1.00	10/10	1.00
R06	10/10	1.00	10/10	1.00	10/10	1.00	10/10	1.00
R07	10/10	1.00	10/10	1.00	10/10	1.00	10/10	1.00
R08	10/10	1.00	10/10	1.00	10/10	1.00	10/10	1.00
R09	10/10	1.00	10/10	1.00	10/10	1.00	10/10	1.00
R10	10/10	1.00	10/10	1.00	10/10	1.00	10/10	1.00
R11	10/10	1.00	10/10	1.00	10/10	1.00	10/10	1.00
R12	10/10	1.00	10/10	1.00	10/10	1.00	10/10	1.00
R13	10/10	1.00	10/10	1.00	10/10	1.00	10/10	1.00
R14	10/10	1.00	10/10	1.00	10/10	1.00	10/10	1.00
R15	10/10	1.00	10/10	1.00	10/10	1.00	10/10	1.00
R16	10/10	1.00	10/10	1.00	10/10	1.00	10/10	1.00
R17	10/10	1.00	10/10	1.00	10/10	1.00	10/10	1.00
R18	10/10	1.00	10/10	1.00	10/10	1.00	10/10	1.00
R19	10/10	1.00	10/10	1.00	10/10	1.00	10/10	1.00
R20	10/10	1.00	10/10	1.00	10/10	1.00	10/10	1.00
R21	10/10	1.00	10/10	1.00	10/10	1.00	10/10	1.00
R22	10/10	1.00	10/10	1.00	10/10	1.00	10/10	1.00
R23	10/10	1.00	10/10	1.00	10/10	1.00	10/10	1.00
R24	10/10	1.00	10/10	1.00	10/10	1.00	10/10	1.00
R25	10/10	1.00	10/10	1.00	10/10	1.00	10/10	1.00
R26	10/10	1.00	10/10	1.00	10/10	1.00	10/10	1.00
S-CVI/Ave		1.00		1.00		1.00		1.00
S-CVI/UA		0.98		0.98		0.98		0.98
